# A Case of Angio-Neurotic Edema, Showing Remarkable Heredity

**Published:** 1893-12

**Authors:** William C. Fritz

**Affiliations:** Resident Staff Buffalo General Hospital


### A CASE OF ANGIO-NEUROTIC EDEMA, SHOWING REMARKABLE HEREDITY.

Reported by WILLIAM C. FRITZ, M. D.,
Of Resident Staff Buffalo General Hospital.

Mr. P., aged 23 ; single ; printer by occupation ; born in United States. Patient was admitted to the Buffalo General Hospital during the service of Dr. W. C. Phelps, May 18, 1893, where he gave the following history :

On May 17, 1893, he fell, striking on his right temple ; shortly after this his left eyelid began to swell, and very soon the eye was completely closed. This swelling extended downward, involving the cheek, lip, and cellular tissue of neck. On entering the hospital the patient was slightly cyanosed, suffering from dyspnea, almost entire loss of voice, and expectorating a moderate amount of frothy material, On physical examination, the uvula was found to be about the size of a hickory nut ; left tonsil, pillar of fauces, and left side of glottis were very edematous. During the following night the swelling began to extend to the right side of the face, at the same time subsiding where it had first appeared. On examination of chest, lungs and heart were found normal.

Next day, May 19, 1893, after some of the acute symptoms had disappeared, the family history was obtained as follows : his paternal grandmother died with edema of the glottis. His father was insane six months before death, the cause of death being abscess of the lungs. His mother is alive and well. One aunt had an edema which appeared and disappeared at various times. Two of her children died with edema of the glottis. Patient had two brothers and two sisters. One sister is comparatively healthy, the other is troubled with edema of the glottis, and both his brothers died with this form of edema. This makes a total of eight persons in the family who have suffered edema.

Patient has always enjoyed good health, but stated that drinking on an empty stomach, overeating, or slight injuries, would bring on attacks of swelling, which usually appeared on extremities and eyelids, and that he had been troubled with this ever since he was four years old.

In conclusion, I will state that the following day the edema of face and neck had subsided, but at the same time his right foot and ankle began to swell, whereupon an examination of his urine was made, and it was found that he passed 900 cc. of urine in twenty-four hours; specific gravity, 1022, acid reaction, containing 28.8 grams of urea; no albumin; no sugar. Microscopical examination showed uric acid crystals and amorphous urates; no casts.

The treatment consisted of hot fomentations and rest. On May 22, 1893, after all signs of edema had disappeared, patient was discharged.

